# Septins in Stem Cells

**DOI:** 10.3389/fcell.2021.801507

**Published:** 2021-12-09

**Authors:** Tanja Schuster, Hartmut Geiger

**Affiliations:** Institute of Molecular Medicine, Ulm University, Ulm, Germany

**Keywords:** septin, Borg, Cdc42, HSC, aging, stem cells, polarity, yeast

## Abstract

Septins were first described in yeast. Due to extensive research in non-yeast cells, Septins are now recognized across all species as important players in the regulation of the cytoskeleton, in the establishment of polarity, for migration, vesicular trafficking and scaffolding. Stem cells are primarily quiescent cells, and this actively maintained quiescent state is critical for proper stem cell function. Equally important though, stem cells undergo symmetric or asymmetric division, which is likely linked to the level of symmetry found in the mother stem cell. Due to the ability to organize barriers and be able to break symmetry in cells, Septins are thought to have a significant impact on organizing quiescence as well as the mode (symmetric vs asymmetric) of stem cell division to affect self-renewal versus differentiation. Mechanisms of regulating mammalian quiescence and symmetry breaking by Septins are though still somewhat elusive. Within this overview article, we summarize current knowledge on the role of Septins in stem cells ranging from yeast to mice especially with respect to quiescence and asymmetric division, with a special focus on hematopoietic stem cells.

## Septins: A Higher-Order Structure for Complex Functions of Cells

The first image of Septins was an electron microscopy image taken in 1976 (Byers and Goetsch, 1976), 6 years after the discovery of Septins in yeast by L. H. Hartwell (Hartwell et al., 1970). It shows the budding of Saccharomyces cerevisiae and examines the neck in cytokinesis which evolves during budding. On the inner side of the plasma membrane 10 nm filaments are highly ordered in a ring-like structure. Yeast has seven different Septins: Cdc11, Cdc12, Cdc3, Cdc10, Shs1, Spr3 and Spr28 (Pan et al., 2007). These Septins form palindromic hetero-octamers in the order: Cdc11/Shs1-Cdc12-Cdc3-Cdc10-Cdc10-Cdc3-Cdc12-Cdc11/Shs1 (Weems et al., 2017). Spr3 and Spr28 are sporulation-specific Septins and can replace Cdc12 and Shs1, respectively (McMurray and Thorner, 2008; Heasley and McMurray, 2015). According to amino acid sequence homology mammalian Septins are separated among themselves in groups (Kinoshita, 2003). 13 different Septins can be found in animals/humans that belong to four groups: Sept3 (consisting of Sept3, Sept9, Sept12), Sept6 (consisting of Sept6, Sept8, Sept10, Sept11, Sept14), Sept2 (consisting of Sept1, Sept2, Sept4, Sept5) and Sept7 (consisting of Sept7). It is evident that Sept7 is a special group as it only has one member. Septins can form hetero-oligomer filaments and the Kinoshita rule states that a Septin of a group can be exchanged by another Septin of the same group within these filaments which do have a canonical order with Sept7-Sept6-Sept2-Sept9, and can form hexamers (Sept2-Sept6-Sept7-Sept7-Sept6-Sept2) and octamers (Sept2-Sept6-Sept7-Sept9-Sept9-Sept7-Sept6-Sept2) (McMurray and Thorner, 2019; Mendonça et al., 2019; Soroor et al., 2021), but other orders of Septin groups were also reported (Sandrock et al., 2011). The hexamers and octamers can interact with each other and form higher-order structures by end-to-end joining or filament pairing by N-termini face (Jiao et al., 2020) or postulated antiparallel homodimeric coiled-coils (Leonardo et al., 2021) resulting in longer and thicker nonpolar polypeptide filaments or rings (Caviston et al., 2003), gauzes (Garcia et al., 2011) and cages (Lobato-Márquez et al., 2021). High-order Septin structures are more stable than other dynamic cytoskeletal polymers (Hagiwara et al., 2011; Bridges et al., 2014) which makes Septins a good choice for acting both as barriers or scaffolds.

Interestingly though, Septins can be organized in multiple types of structures, implying a versatile but also likely a cell type-specific organization of Septins to serve a special purpose within a cell. Septins show spot-like and blob-like distributions within cells under certain conditions (isolated BD3 domain expression of Borg3 (Joberty et al., 2001)) and in certain cell types as well (Kandi et al., 2021) which are likely not linked to fibers. The ring structure of Septins is associated with cytokinesis in yeast, but Septin rings are also formed around internalized bacteria upon infection of mammalian cell lines with *Listeria* and *Shigella* bacteria (Mostowy et al., 2009) and intra-cytosolic *Shigella* are compartmentalized in Septin cage-like structures for autophagy as a host defense (Mostowy et al., 2010). There is a functional interdependence between Septin and Actin cytoskeleton resulting in O- and C-shaped rings as well as bundles of Septins (Schmidt and Nichols, 2004), and Septins can co-align with microtubules to form bundles (Targa et al., 2019). Arc-shaped (together with ring-like) co-localizing clusters of Sept7, Sept5 and Sept11 could be found in neurons at the base of dendritic spines with the function of building a diffusion barrier for membrane proteins (Xie et al., 2007; Ewers et al., 2014). While Septins are known for their ability to break symmetry within cells (Spiliotis and McMurray, 2020), little is known about the role of Septins in mammalian stem cells. They are though designated candidates for being involved in the organization of symmetry and polarity and thus function of stem cells.

The process of blood cell formation (which is termed hematopoiesis) depends on hematopoietic stem cells (HSCs). Roughly 75% of HSCs are in the quiescent G0 state, about 20% are in G1, <2% can be found in an active-cycling state (G2/S/M) (Cheshier et al., 1999; Wilson et al., 2008). Symmetry in quiescent, resting HSCs has been defined by the distribution of polarity proteins and nuclear markers within HSCs (Florian and Geiger, 2010; Grigoryan et al., 2018). In addition, the extent of this symmetry correlates with the age of the HSCs. With respect to the distribution of Septins within HSCs, recent published data show that both spots and blobs of certain Septins (Sept7, Sept6, Sept2) are found within HSCs (Senger et al., 2017; Kandi et al., 2021b, see Table1). The distinct roles of these specific structures (spots, blobs) remain elusive.

**TABLE1:** Septins in HSCs.

Septin type	Phenotype in HSCs	Murine HSC modification
Sept1 Ni et al. (2019)	Sept1 phosphorylation elevated, impaired cytoskeletal remodeling, decreased cell rigidity, stem cell egress from niche, HSCs with enhanced mobility, decreased quiescence, increased apoptosis and defective reconstitution capacity	Ptpn21^−/−^ HSCs
Sept6 Senger et al. (2017)	Increased engraftment of Sept6^−/−^ HSCs upon transplantation, decreased contribution to T cells and increased contribution to B cells, no influence on cell cycle kinetics, cell cycle status, homing, stress tolerance	Sept6^−/−^ HSCs
Sept7 Kandi et al. (2021)	Sept7 polarity is regulated by Cdc42 activity; regulation of Cdc42-Borg4-Sept7 interaction in HSCs by Cdc42 activity levelSept7^−/−^ and Borg4^−/−^ HSCs present impaired function upon transplantation and show changed Cdc42 distribution Sept7^−/−^ HSCs: impaired proliferation, loss of HSC and progenitor potential upon cell division	Control HSCsSept7^−/−^ HSCsBorg4^−/−^ HSCs

**FIGURE 1:**
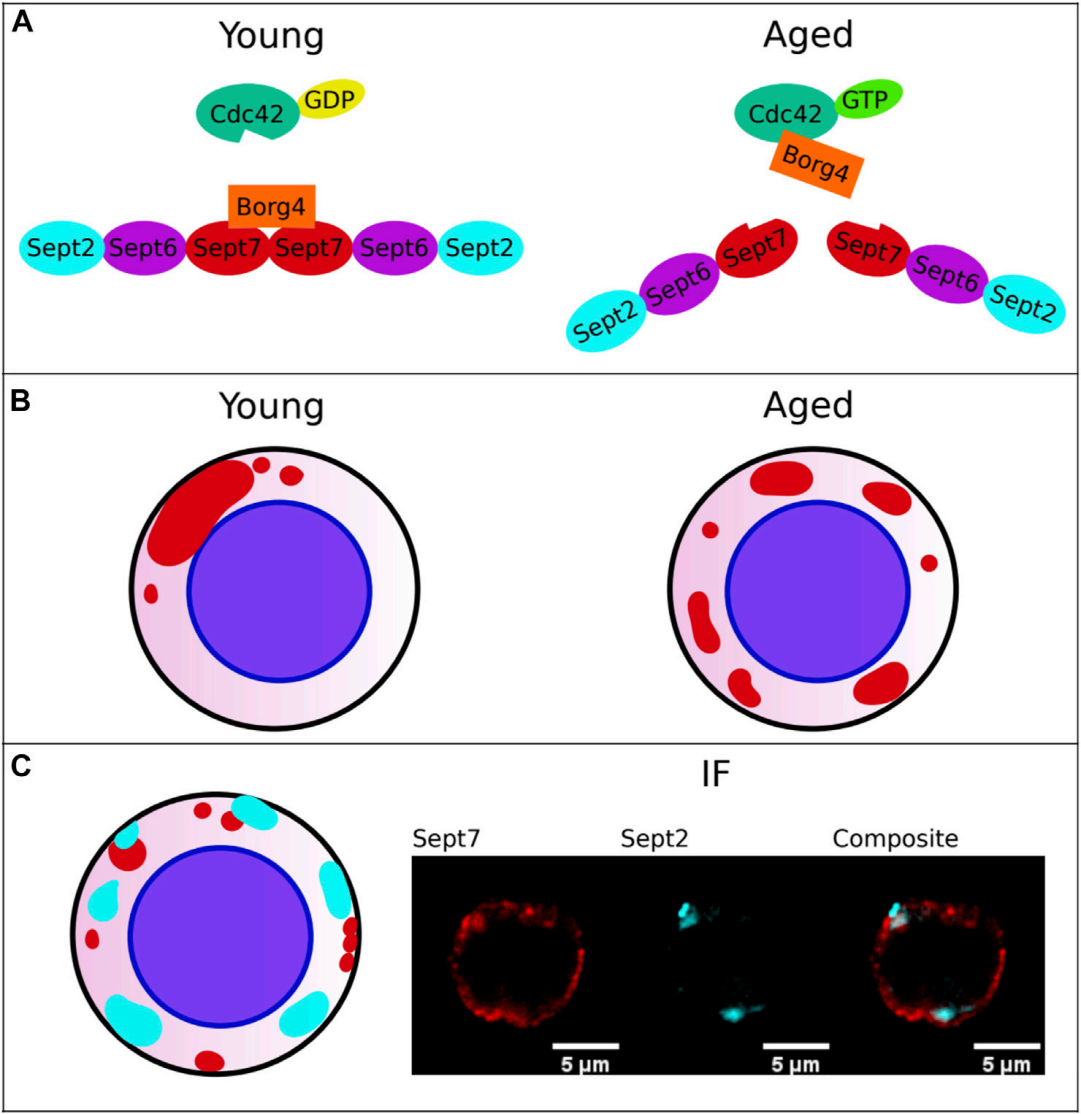
Septin behavior in hematopoietic stem cells (HSCs). **(A)** Change in Cdc42-Borg4-Sept7 axis upon aging. Interaction of Borg4 with Sept7 is shifted upon aging according to elevated activity level of Cdc42 to interaction of Cdc42 with Borg4. **(B)** Change in protein distribution of Sept7 upon aging: Elevated Cdc42 activity leads to a more apolar distribution. **(C)** Interaction of Septins among themselves. Sept7 and Sept2 don’t show same distribution. The Septin Network is more complex and demands further studies.

In young HSCs (from mice that are 4 months old) there is a polar distribution of the polarity proteins RhoGTPase Cell division control 42 protein (Cdc42), Histone 4 Lysine 16 acetylation (H4K16ac), Per2, Numb, Tubulin and also Sept7, with usually one big blob at one spot with perhaps several smaller spots, while in aged HSCs (from animals 18 months and older), this distribution is apolar: more smaller spots, and a much more dispersed distribution (Figure 1B). It is thought that the level of polarity is linked to the mode (symmetric/asymmetric) of stem cell division to balance self-renewal versus differentiation (Florian et al., 2018) and to regulate the homing capability to allow for successful immune system reconstitution after transplantation (Liang et al., 2005; Janzen et al., 2006; Florian and Geiger, 2010; Dykstra et al., 2011; Florian et al., 2012; Sun et al., 2014; Kandi et al., 2021).

Upon aging, the activity of the small RhoGTPase Cdc42, in itself also a polarity protein, is elevated in almost all tissue analyzed so far (Yang et al., 2007b; Florian et al., 2012). This elevated activity of Cdc42 causes a reduced frequency of polar HSCs among aged HSCs. Cdc42 can bind to the effector protein group binders of Rho GTPases (Borgs), which themselves were shown to interact with Septins (Joberty et al., 2001; Sheffield et al., 2003). However, it is neither completely understood how the organization and reorganization of HSCs occurs with respect to Cdc42 and Borgs, nor how the Septin network behaves and changes upon aging in HSCs.

## Decipher Septin Contribution to Mammalian Stem Cells and Aging via Yeast

### Yeast as Role Model for Stem Cells?

As there is more information on Septins in yeast, findings in yeast might inform on Septin biology also in mammalian HSCs. This will help in formulating open questions that need to be addressed in the future. Yeast possesses characteristic traits of stem cells: quiescence and asymmetric division (Werner-Washburne et al., 1993; Gray et al., 2004; Higuchi-Sanabria et al., 2014). In the past, yeast has already successfully been taken as a comparison model for metabolic changes in quiescence entry and exit (Dhawan and Laxman, 2015). Replicative life span and chronological life span can be straightforward examined in yeast (Postnikoff and Harkness, 2014). Elevated activity of Cdc42 is linked to aging in HSCs and to replicative lifespan in yeast (Kang et al., 2021). Thus, yeast is both a good stem cell and aging model from which processes involved in impairing functions upon aging could be translated into HSCs. This could lead to clinical translations in improving transplantation success and immune system.

On the other hand, there are of course also distinct roles for Septins in yeast and HSCs. Septins for example are indispensable for yeast division (Hartwell et al., 1970), yet especially for the hematopoietic system it was shown that cytokinesis may occur without Septins, while Septins are indispensable for cytokinesis in fibroblasts (Menon et al., 2014). Novel anti-Septin cancer drugs for solid tumors might thus benefit from the fact that Septins are dispensable for hematopoietic cytokinesis while being critical for cancer cells (Menon and Gaestel, 2015). In summary, published data imply that while yeast can serve as a stem cell model and inform on roles of Septins in HSCs, Septins also show a cell type-specific behavior, which requires a careful approach when translating findings in yeast to HSCs.

### Quiescence

Quiescence is a cell state in which cells exit the cell cycle and rest in a more robust, withstanding state (G0) till they respond to a signal and reenter the cell cycle. Features of quiescence spanning across species are: reduction of cell size, arrest of cell cycle, condensation of chromosomes, reduction of rRNA synthesis and reduction of protein translation, increase in autophagic activity and increase in stress resistance (Hendil, 1981; Kniss and Burry, 1988; Nagele et al., 1999; Kops et al., 2002; Yusuf and Fruman, 2003; Zhang et al., 2003; Gray et al., 2004; Dhawan and Laxman, 2015; Roche et al., 2017; Wang et al., 2017; Sun and Gresham, 2021).

Many types of extrinsic and intrinsic signals maintain HSCs in quiescence (Liu et al., 2009; Matsumoto et al., 2011; Yamazaki et al., 2011; Nakamura-Ishizu et al., 2014).

As HSC quiescence entry and exit is dependent on the conversion of the cytoskeleton, changes in the localization and structure of organelles will thus likely depend on the well-known properties of Septins like organizing, scaffolding and barricading. Currently, there is no direct data on the role of Septins upon HSCs leaving or entering the quiescent state. The role of Septins in quiescent yeast cells might thus help to inform on likely roles of Septins in quiescent mammalian HSCs.

Yeast enters the quiescent state upon nutrient deprivation or desiccation and several pathways like Target of rapamycin complex I (TORC1), Sucrose non-fermenting 1 (SNF1) protein kinase, Protein kinase A (PKA) and Pho80-Pho85 (Forget et al., 2002; Sopko et al., 2007; Xu et al., 2012) all contribute to this decision. Downregulation of TORCI, a downregulation of PKA, a downregulation of Pho80-Pho85 or an upregulation of SNF1 results in entering of the quiescent state in yeast. These signals are, at the end, all integrated and feed into the regulation of the activity of Cdc42. For mammalian cells similar pathways have been reported for being responsible for the regulation of quiescence in HSCs (Chen et al., 2008; Gan et al., 2010; Cheng et al., 2014), and it was shown that Cdc42 is important for quiescence in HSCs, as the Cdc42 knockout mouse model, which though does not represent physiological Cdc42 deviations upon aging, results in HSC cycling (Yang and Zheng, 2007a).

These findings lead to the following questions with respect to the role of Septins in quiescence: 1) How is Cdc42 linked to Septins? 2) How does Cdc42 affect Septins? and 3) Do interactions of Septins with other cytoskeletal proteins change upon quiescence?

1) A whole family of interaction partners linking Cdc42 to Septins was found in 1997: Gic1 and Gic2 bind specifically to Cdc42-guanosine triphosphate (Cdc42-GTP) (Brown et al., 1997) as well as to Septins (Sadian et al., 2013). It was shown that Gic1 behaves as a scaffolding protein for Septin filaments and Cdc42-GTP binding to Gic1 results in dissociation of Gic1 from Septin filaments. Cdc42-GDP can directly disassemble Septin filaments in yeast when Gic1 is missing. A functional mammalian equivalent was found in 1999 by Joberty et al.: Borgs (Binder of Rho GTPases) 1-5, also entitled as Cdc42 effector proteins 1-5, bind Cdc42 in a GTP-dependent manner (Joberty et al., 1999). HSCs do also express Borg proteins (Kandi et al., 2021). Among the above mentioned pathways linked to quiescence, also Iqg1 of the yeast TORC1 pathway was shown to be involved in Septin ring organization and regulation of Cdc42 activity (Iwase et al., 2007; Atkins et al., 2013). The mammalian homologue IQGAP1 binds the Exocyst-Septin complex via its N-terminus, probably via Sept2, and Cdc42-GTP via its C-terminus (Hedman et al., 2015; Wang et al., 2009) showing functional interdependence like regulation of secretion (Rittmeyer et al., 2008). Normal levels of Cdc42 activity (more GDP) regulate the assembly of Septin fibers via “free to bind Borgs”, and Borgs lead to a stabilization and bundling of Septin filaments, which is consistent with the strong focal assembly (big blob) of Septins seen in young HSCs (Figure 1B). Such a view is further consistent with the recent finding from our laboratory that upon aging of HSCs, there is a switch in the Cdc42-Borg-Septin relationship: Elevated Cdc42 activity in aged correlated with co-localization of Cdc42 and Borg4, whereas in young HSCs (lower Cdc42 activity) Borg4 and Sept7 co-localized (Kandi et al., 2021) (Figure 1A). In conclusion, these data support that Cdc42 activity influences Septin behavior in both yeast and adult stem cells like HSCs and might also be critical for the role of Septins in quiescence.

2) Cdc42 activity changes upon quiescence as quiescence requires a downregulation of mTOR, of PKA, of Pho80-Pho85 and an upregulation of Snf1/AMPK, which altogether means more Septin-Exocyst complexes being bound to Iqg1 (mTOR, Snf1) (Xu et al., 2012), a dampened activity of Cdc42 (Pho80-Pho85) (Paglini et al., 2001; Sopko et al., 2007) with more membrane-bound Cdc42 (PKA) (Forget et al., 2002; Johnson et al., 2009). In summary, there is indeed a switch of Septin-Gic/Borg interactions regulated by Cdc42 activity in yeast and HSCs, which influences bundling and stabilization of Septin filaments. As it has not been tested though whether Cdc42-GDP indeed disturbs Septin filaments in HSCs like it does in yeast, it still remains a possibility that the Cdc42-Borg-Septin axis is somewhat distinct between yeast and HSCs with respect to quiescence.

3) While Septins also actively participate in forming the cytoskeleton (Kinoshita et al., 2002; Mavrakis et al., 2014; Kuzmić et al., 2021), they are not directly involved in the reorganization and restructuring of Actin and microtubules upon quiescence in yeast (Sagot et al., 2006; Laporte et al., 2013; Laporte et al., 2015). However, in HSCs a link of Septins to the cytoskeleton was shown by the finding that Ptpn21 localizing to Actin filaments is important for Sept1 dephosphorylation and Sept1 dephosphorylation level is crucial for HSC stiffness and retention in the bone marrow niche (Ni et al., 2019 and see Table1). Thus, in this case, investigations into the role of Septins in HSCs might inform on likely mechanisms of interactions of Septins with the cytoskeleton in yeast. Nonetheless, the involvement of Septins in reorganization of the other cytoskeleton components upon quiescence is still uncharted territory. An in depth understanding of the role Septins play in regulating quiescence will be important for basic stem cell biology, aging and cancer research.

### Asymmetric Division

Asymmetric divisions first allow stem cells to provide a more differentiated cell that will contribute to tissue homeostasis while maintaining the pool of stem cells. Asymmetric division is also thought to be the key to produce one daughter cell which is free of already damaged products and one daughter cell which takes all these damaged products, and is regarded as kind of a rejuvenation strategy (Higuchi-Sanabria et al., 2014). Several organelles, proteins and mRNAs are asymmetrically inherited to daughters in yeast (McFaline-Figueroa et al., 2011; Shcheprova et al., 2008; Kennedy and McCormick, 2011; Henderson et al., 2014; Yang et al., 2015; Spokoini et al., 2012). The same holds true for mammalian stem cells (Inaba and Yamashita, 2012; Katajisto et al., 2015; Wang X. et al., 2009; Paridaen et al., 2013; Kuo et al., 2011; Rujano et al., 2006; Fuentealba et al., 2008; Loeffler et al., 2019; Hinge et al., 2020; Vannini et al., 2019; Loeffler at al., 2021). Proteins and organelles need to be distributed in a polar fashion in cells during mitosis to allow for an asymmetric split-up. Our data show that polarity/symmetry in quiescent cells correlates with the type of division mode (symmetric/asymmetric) (Florian et al., 2018). Similar findings were made for mitochondria inheritance in HSCs (Hinge et al., 2020) and inheritance of lysosomes, autophagosomes, mitophagosomes, NUMB, Notch1 and CD63 which functionally predict the activation of daughter cells and thus their fates (Loeffler et al., 2019).

As Septins are master regulators of asymmetry and polarity (Spiliotis and McMurray, 2020), it is very likely that they play a role not only in the asymmetric division of budding yeast but as well also in HSC division and general asymmetric distribution of proteins within HSCs. We could recently demonstrate that Sept7 is indeed involved in regulating the distribution of polarity proteins in HSCs (Kandi et al., 2021, see Table1). Budding yeast achieves polarity and thus asymmetry during cell cytokinesis by three consecutive steps with enduring Septin support: 1) deciding upon a bud site and 2) passage of cellular components through a barrier and 3) active retainment of components within a compartmentalized space.

1) The first step is made by landmark proteins that then activate Rsr1 at a specific location. Rsr1-GTP recruits Cdc24, which functions as guanine-nucleotide-exchange factor (GEF) for Cdc42. Cdc42 is activated by Cdc24. Cdc42 further accumulates by positive feedback mechanisms that result in an area of high density of Cdc42-GTP. Septins then move to the high density Cdc42-GTP area. Right before budding Cdc24 interacts with the Septin Cdc11, which is essential for Septin recruitment and stability of the polarity patch (Park et al., 1997; Kang et al., 2001; Shimada et al., 2004; Chollet et al., 2020). Septins then form the characteristic ring-like filaments at the bud site (Okada et al., 2013). Cdc42 activity therefore plays a role in the polarization of the Septin ring. Whether there is a ring-like structure of Septins upon initiation of division of HSCs is currently not known.

2) The second step is regulated by the Septin ring which functions like a lateral diffusion barrier and compartmentalizes the bud. Septins can via this means function as a barrier for misfolded proteins of endoplasmic reticulum (ER) in the mother cell in a Septin-, Bud1-and sphingolipid-dependent manner (Chao et al., 2014; Clay et al., 2014). Similar mechanisms are proposed for the role of Septins in neurons, in which dendritic spines are restricted in the diffusion of ER proteins (Tada et al., 2007; Xie et al., 2007). It is thus likely that Septins can also play a similar role in HSCs, and also function in HSCs both as scaffold and as barrier in the process of asymmetric divisions.

3) The third step, active retainment, is fulfilled by anchor proteins that are transported to the bud and retain transported organelles and proteins in the bud or in the mother cell. While asymmetrically inherited mRNA is transported by Actin (Munchow et al., 1999; Takizawa et al., 2000), Septins act like a diffusion barrier by holding proteins that anchor organelles to the bud tip like Mmr1. (Fehrenbacher et al., 2004; McFaline-Figueroa et al., 2011; Swayne et al., 2011; Higuchi et al., 2013). Septins were also shown to interact with membrane components like phosphatidylinositol-4,5-bisphospate (PIP2). Septins are curvature-sensitive and capable of shaping the form of membranes (Gladfelter et al., 2005; Tanaka-Takiguchi et al., 2009; Bertin et al., 2010; Beber et al., 2019; Cannon et al., 2019). It is thus a possibility that Septins form lipid scaffolds for proteins, which themselves do not interact with Septins but are retained due to specific lipid or protein composition of the lipid scaffold. Research into the interaction of the lipidome with Septins will be necessary to obtain novel information on this likely novel role of Septins in the regulation of asymmetry, also presumably in HSCs.

While there has been ample progress in understanding the role of Septins for asymmetric division in yeast, so far, there have been no investigations published on the role of Septins and the Septin network in HSCs or even other stem cells for retaining or repulsing proteins and organelles upon division. Mammalian stem cell research has been focused on the role of individual Septins and their role for organelles. A disruption of mitochondrial fission in human mammary stem-like cells leads to symmetrical inheritance (Katajisto et al., 2015). A Drp1-mediated, distinct localization of old mitochondria is important for this asymmetric inheritance (Hinge et al., 2020). Sept2 is a known Drp1 interaction partner involved in mitochondria fission, implying indeed that Sept2 might be involved in an asymmetric inheritance of mitochondria upon division (Pagliuso et al., 2016). Centriole and cilium inheritance is asymmetric in radial glial progenitor cells (Wang X. et al., 2009; Paridaen et al., 2013). As Sept7 is a centrosomal protein and is involved in the process of mitotic spindle pole formation (Chen et al., 2021), it is possible that it also plays a role in asymmetric inheritance of centrioles and primary cilium in progenitor cells. A main unsolved question remains the nature of the role of the Septin network and thus filaments in HSCs and even in yeast. While the function of Septins has been frequently linked to their function within the filament network, recent data from our laboratory imply a non-filament linked, distinct polar/apolar distribution of Sept7, Sept2 and Sept6 in HSCs (Kandi et al., 2021b). For example, the distribution of Sept7 and Sept2 in HSCs is quite distinct (Figure 1C), which questions whether these Septins are indeed strongly interacting. Novel research on the role of Septins for the division of HSCs will thus need to focus on both the role of the Septin network as well as the role of individual Septins.

## Perspectives

While Septins have been investigated since the early 1970s, even after now more than these 50 years of Septin research, the Septin field is still an emerging field with recent novel exciting developments like a revisited order of human Septins (instead of the previously postulated hexamer form “Sept7-Sept6-Sept2-Sept2-Sept6-Sept7” the novel hexamer form reads “Sept2-Sept6-Sept7-Sept7-Sept6-Sept2” and instead of the postulated octamer form “Sept9-Sept7-Sept6-Sept2-Sept2-Sept6-Sept7-Sept9” the novel octamer form reads “Sept2-Sept6-Sept7-Sept9-Sept9-Sept7-Sept6-Sept2”). (McMurray and Thorner, 2019; Mendonça et al., 2019; Soroor et al., 2021). So far, little is known about the role of Septins in maintenance of quiescence in yeast and stem cells, while the same holds true for the role of Septins in asymmetric division and asymmetric protein distribution in stem cells. The role of Septins for an asymmetric division in yeast has been extensively studied, and in general, Septin biology in yeast might thus serve indeed as an initial blueprint for Septins in other types of stem cells like HSCs.

It is becoming more and more obvious though that Septins act also very cell type-specific, and have specific functions in distinct types of cells, especially among differentiated cells or cell lines. It will become therefore critical to investigate the role of Septins in a variety of cell types in order to understand their distinct behavior and to initiate transition of knowledge for example to drug development approaches to harvest differential sensitivities of cells to drugs targeting specific Septins (Menon and Gaestel, 2015). Another critical question that requires more attention is the organization state of Septins within cells, as both Septin filaments but also individual Septins outside of filaments are likely to have major biological function. Novel proteomics, lipidomics and immunofluorescence approaches that work on a few or even single cells will support the hunt for interaction partners of Septins and the organization of the Septin network in rare stem cells. It is likely that stem cells, due to their requirement of balancing quiescence and cycling and symmetric and asymmetric division might provide a fertile ground for elucidating these complex and context-dependent roles already within 1 cell type, as the underlying complexity that requires a versatile function of Septins might likely not be found in differentiated cells.
